# Developing a patient safety guide for primary care: A co‐design approach involving patients, carers and clinicians

**DOI:** 10.1111/hex.13143

**Published:** 2020-11-03

**Authors:** Rebecca L. Morris, Angela Ruddock, Kay Gallacher, Carly Rolfe, Sally Giles, Stephen Campbell

**Affiliations:** ^1^ NIHR Greater Manchester Patient Safety Translational Research Centre Faculty of Biology, Medicine and Health Division of Population Health Health Services Research and Primary Care School of Health Sciences University of Manchester Manchester United Kingdom

**Keywords:** co‐design, health care professional experience, improvement, patient experience, patient safety, primary care, UK

## Abstract

**Background:**

Patients and carers should be actively involved in patient safety and empowered to use person‐centred approaches where they are asked to both identify safety concerns and partner in preventing them.

**Objectives:**

The aim of this study was to co‐design a patient safety guide for primary care (PSG‐PC) to support patients and carers to address key patient safety questions and identify key points where they can make their care safer. The objectives were to i) identify when and how patients and carers can be involved in primary care patient safety, and ii) identify the relevant information to include in the PSG‐PC.

**Design:**

An experience‐based co‐design approach.

**Setting and Participants:**

We conducted three workshops with patients, carers, community pharmacists and general practitioners to develop and refine the PSG‐PC.

**Results:**

Participants identified both explicit and implicit issues of primary care patient safety especially relating to technical and relational components of involving patients and carers. The importance of communication, understanding roles and responsibilities, and developing partnerships between patients and health‐care providers were considered essential for actively involving patients in patient safety. Co‐developing the PSG‐PC provided insight to improve care to develop the PSG‐PC.

**Discussion:**

The PSG‐PC is the first guide to be developed for primary care, co‐designed with patients, carers, general practitioners and pharmacists. The PSG‐PC will support patients and carers to partner with health‐care professionals to improve patient safety addressing international and national priorities to continuously improve patient safety.

## 1 INTRODUCTION

Globally health‐care systems are experiencing unparalleled levels of demand for services, and a greater focus on long‐term condition management as well as financial constraints and an increasing role of technology in care. Improving the quality and safety of health care is a key priority.^1^, ^2^, ^3^, ^4^ There has been an increasing focus on patient safety globally especially on preventing the most common causes of harm such as prescribing, diagnosis and treatment in primary care (ie the first point of contact in the health‐care system typically including in the United Kingdom (UK) General Practice and community Pharmacy).^1^, ^4^, ^5^, ^6^ Patient safety is ‘the avoidance, prevention and amelioration of adverse outcomes or injuries stemming from the processes of health care’. ^7^ For example, in primary care diagnostic and medication incidents were most likely to result in harm or severe harm. Patient‐provider communication issues also contribute to patient safety incidents either directly or indirectly.^2^, ^8^ There has been a shift from a medico‐legal and blame‐focused approach towards a focus on understanding and learning from incidents.^9^, ^10^ However, there often remains a focus on accountability rather than embedding a systems based learning approach that includes patients and carers.^9^, ^10^, ^11^

In the UK, national reports about patient safety have recommended that patients should be involved at all levels of their patient safety in an open and transparent way with accurate and useful information.^12^, ^13^ The National Patient Safety Strategy has emphasized that improving patient safety requires a dual focus on learning from events and lived experience to prevent harm whilst also anticipating, predicting and preventing unsafe care/services, which requires action ranging from government, health‐ and social care staff and patients and carers.^14^ In essence patient safety can be defined as the ‘avoidance, prevention and amelioration’ of adverse outcomes and harm in health care, the ‘avoidance, prevention and amelioration’ of adverse outcomes and harm in health care.^7^, ^14^, ^15^ This is equally a key element of the UK national patient safety syllabus but despite this and increasing focus on patient involvement in patient safety, in primary care there remains a gap as to how to practically support patients with national guidance identifying tools in secondary care but not in primary care.^14^, ^15^ Furthermore, whilst much of the research focus for patient safety has been on secondary care, the volume of patient contact with primary care makes patient safety initiatives in this setting a priority.^16^, ^17^, ^18^, ^19^, ^20^, ^21^

Many of the mechanisms for improving patient safety focus on health‐care professionals such as systems to track and report errors, regulations and accreditation and work to engage doctors and nurses with patient safety.^22^, ^23^, ^24^, ^25^, ^26^, ^27^, ^28^ However, patient and health‐care professional perceptions’ of safety differ. Patients place a greater emphasis on a wider psychosocial context of safety (eg feeling listened to and able to raise concerns).^9^, ^18^ Patients tend to view both serious and relatively not as serious issues which cause distress as psychological safety concerns.^28^ Much of the research on patient safety in primary care is descriptive, with few of the studies focusing on interventions that will improve it that supports patients in primary care with medication, information exchange to diagnosis and treatment across care.^2^, ^29^, ^30^, ^31^, ^32^, ^33^ Primary care work often includes managing uncertainties (eg symptoms presenting potential differential diagnoses) along episodes of care, often over a long period, for example uncertainty in diagnosis, monitoring, and treatment, or providing self‐management support which creates a different context for care than secondary care and may require different ways of identifying and improving patient safety.^32^, ^33^, ^34^, ^35^, ^36^, ^37^ Furthermore, overdiagnosis may also cause harms and distress.^33^, ^38^

Areas that can contribute to enhancing safety, beyond the prevention of serious incidents, involve patients include patient‐professional communication, the role of intermediaries and collective‐level forums.^39^ Defining and applying strategies to improve patient safety involve an on‐going dialogue between patients and health‐care professionals^6^ that build on patient‐centred approaches to care developing trust, clarify expectations and ensure understanding.^29^, ^30^ The timing, authenticity and ability to discuss experiences with clinicians are key elements for patients speaking up when patients have concerns about their care if they wish to do so.^16^ In parallel, tools developed to support patients and carers to be involved in patient safety (if they want) need to have appropriate information that is open, transparent, accurate and useful.^12^, ^13^ This approach also reflects the wider shift in recognizing patients as an additional safeguard within the health‐care system, which aligns with a systems approach to patient safety.^30^, ^31^, ^32^

A patient's understanding of safety and the health‐care system in which it occurs is interdependent.^1^ Patients who may wish to raise safety concerns may be vulnerable to or perceive a power imbalance that may influence the willingness or ability of the patient to raise patient safety issues if they would like to.^39^, ^40^ There are a number of factors that may influence a patient's willingness to be involved. These can be patient‐related, illness‐related, health‐care professional‐related, setting‐related and task‐related.^41^, ^42^ Involving patients in patient safety requires a collaborative approach as there are multiple interacting influences on safety and solutions to address issues.^43^ One way that has been proposed to understand these multiple interacting influences on safety to develop support for patients and carers is to bring patients, carers and health‐care professionals together to develop initiatives that do not disenfranchise or overburden any one stakeholder. This approach can move research findings into practical, meaningful outcomes that incorporates forms tacit, behavioural and experiential knowledge.^44^

Whilst there are handbooks and tools developed for patients to support their involvement in safety,^45^, ^46^, ^47^ the role of involving patients actively in patient safety remains whilst increasing remains underexplored in primary care. These tools have provided a comprehensive support for patient safety in secondary care (including safety tips, sections for personal information, treatment plans, and information on specific issues such as hygiene and fall prevention) but an equivalent version does not exist in primary care.^46^ In accordance with the Medical Research Council Framework for the design and evaluation of complex interventions, this paper reports the first phase in the programme of work to develop and test the patient safety guide for primary care (PSG‐PC).^48^ The aim of this study was to develop the PSG‐PC to support patients and carers to identify key patient safety issues and identify key points where they can make their care safer in primary care, co‐designed by patients and carers and health‐care professionals to support their involvement in primary care patient safety and to ensure the content of the PSG‐PC is acceptable and accessible.

## 2 METHODS

The development of the PSG‐PC took place between May 2015 and July 2019. It involved a cyclical approach based on the experience‐based co‐design approach.^49^, ^50^ The approach used existing evidence of primary care patient safety experiences from the wider literature to inform the initial trigger discussion and then followed by a series of workshops and prototype development in an iterative and cyclical approach to development.

Experience‐based co‐design (EBCD) is one form of narrative‐based, participatory action research.^51^, ^52^, ^53^ It has been defined as a form of ‘user‐focused design process with the goal of making user experience accessible to the designers, to allow them to conceive of designing experiences rather than designing services’.^51^ EBCD requires collaboration between all stakeholders focusing on user experiences (both on a cognitive and emotional level) through identifying key touch points and working together in co‐design events to identify where experiences can be improved.^51^, ^53^ These touch points reflect a significant experience which creates a strong emotional response and may reflect a core element of health care.^54^, ^55^ EBCD has been used for quality improvement initiatives in a variety of settings, such as emergency medicine, primary care and mental health services to make changes to improve care.^52^, ^53^, ^56^ In co‐design, user narratives (including stories and scenarios) may be used to communicate ideas and concepts and how they might be used.^53^ This approach can start with a broad brainstorming process where all dimensions of a problem can be considered, and then a narrowing of focus to identifying potential solutions which have can for all stakeholders.^54^

### 2.1 Initial set‐up: Establishing patient and public, and health‐care professional, stakeholder groups

Involving members of the public in the design and development of the project was key from the onset to ensure that research is not just to done ‘to’ or ‘about’ people but is being carried out ‘with’ or ‘by members of the public’.^57^, ^58^ The initial PSG‐PC project was presented to a patient and public involvement group (whose aim was to offer advise to research, comment on and develop research materials and work with researchers as a member of the public or person with a lived experience as a service user) within the [NIHR Greater Manchester Patient Safety Translational Research Centre (NIHR GM PSTRC)].^58^ Six public contributors (ie the preferred role title by members of the patient and public involvement group) volunteered to work with [RM] the research team and as part of a patient and public involvement group. The patient and public involvement group met regularly throughout the study working with [RM] to design the study, develop ideas, plan and run the co‐design workshops, developing and refining the PSG‐PC prototypes, and promoting and disseminating the study. Concurrently, a virtual health‐care professional stakeholder (which is a group of general practitioners (GPs) or community pharmacists who commented on the research from a health‐care professional perspective) and public contributor groups were established. GP and pharmacists were included as they provide the majority of primary care services in the UK.^59^ Members were recruited to these groups through a range of adverts (online via twitter, the [department] external newsletter and snowball recruitment through existing contacts). All public contributors throughout the study were reimbursed for their time at INVOLVE (ie a national advisory group for patient and public involvement in the NHS, public health‐ and social care research in England) rates,^60^ and all health‐care professionals were reimbursed for pro‐rata of their standard payment rates. The groups worked with the research team to identify key touch points for primary care patient safety (eg access, diagnosis and medication) and develop the first co‐design workshop.

### 2.2 Participant recruitment

Patients, carers, GPs and pharmacists were recruited to be involved in the study as users of primary care services (ie patients and carers) and as the main health‐care professionals delivering primary care services in the UK (ie GPs and pharmacist). The inclusion criteria were either to be a patient, carer, practising GP or community pharmacist over the age of 18 able to understand and communicate sufficiently in English to participate. Participants were recruited through online adverts, twitter, GMPSTRC external newsletter and through existing contacts. Due to the nature of this type of broad advertizing, it is unknown how many people choose not to participate. The adverts were available online and so potentially anyone in the UK could have attended, however, as these were meetings held on one day in North West England for logistical constraints participants were from the North West England area.

### 2.3 Co‐design process

#### 2.3.1 Initial co‐design workshop

The first co‐design workshop was held in March 2016. Participants included a mix of patients, carers general practitioners (GP) and community pharmacists. The workshop was categorized as a public involvement workshop by the Health Research Authority definition and discussions were not being videoed or audio recorded so participants were not asked to sign consent.^61^ As this was part of a wider study, this approach has been given ethical approval (research ethics committee reference: 16/YH/0496).

A morning introduction session outlined the approach [RM and SC] and introduction of NIHR GM PSTRC, and a presentation was given to trigger discussion about an experience of a family member trying to access primary care services by a member of the public contributor group [KG and AR]. The day was divided into two facilitated discussions, which aimed to:

1. Identify key patient safety questions and identify key touch points where patients can be involved in patient safety from the patients, carers and staff perspectives.
2. Identify the information and practical ways of improving this that could be included in a PSG‐PC.

**FIGURE 1:**
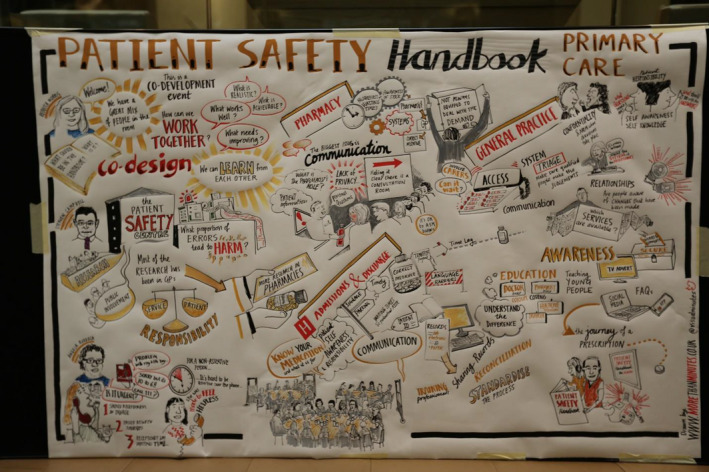
Visual minutes made of the discussion from Workshop 1

Participants were asked to brainstorm their initial thoughts and write them on post‐it notes and then in smaller groups held a facilitated discussion to identify the patient journey in primary care (eg going to the GP) and the key touch points that they identified. After the initial facilitated discussion about these touch points, participants were then asked to identify the actions that could realistically be done to improve them. Each small group fed back to the whole group the key touch points and actions to improve them that they identified. Using a consensus approach at the end of each session, participants were asked to vote on main points. Each group was facilitated by at least one public contributor from the public involvement group and a health services researcher. After this session, briefing notes were sent to all facilitators. Facilitators collected information from participants (eg through post‐it notes, diagrams) and recorded field notes from individual group discussions. [RM and SG] also took field notes during the workshop, and visual minutes were created during the day (see Figure 1). These notes were then analysed thematically to identify key themes within the data (see below for more details).^62^ A summary of the workshop and themes was sent to all participants to ensure that participants’ accounts were presented accurately.

#### 2.3.2 Prototype development and refinement

A prototype of the PSG‐PC was developed. This used the notes and feedback from participants on the key components for the PSG‐PC. This consisted of the key touch points of contact between patients and carers with primary care services and what was identified as working well and what could be improved.^54^, ^63^ The development was an iterative process involving discussions with the public contributors, health‐care professional contributors and the wider research team via a combination of group meetings and email discussions. Additional co‐design workshops were held in March 2017 and 2018 to get feedback on the PSG‐PC prototype and how the PSG‐PC could be realistically implemented into everyday practise. Participants were given the PSG‐PC to look at on the day so that real‐time feedback and then worked with participants to interpret their feedback and to consider the key touch points initially identified that captured a core challenge of involving patients and/or carers in patient safety in primary care.^54^ There were three groups facilitated by public contributors as well as a health services researcher, and notes were recorded and analysed. Post‐workshop feedback was collected via a short online survey, and participants were given the opportunity to feedback any additional comments via email to ensure interpretation was accurate. The PSG‐PC prototype was refined working with the patient and public, and health‐care professional, stakeholder groups following the co‐design events. The PSG‐PC was then refined based on these discussions with feedback from representatives from national organizations with a focus on patient safety. The PSG‐PC was awarded the Plain English campaign Crystal Mark to ensure the PSG‐PC was clear and concise.^64^

The reporting of this study has adhered to the GRIPP 2 checklist to enhance the quality and transparency of patient and public involvement reporting.^65^

### 2.4 Data collection

Data from the co‐design events were collected and used to identify key touch points of patient experiences to identify key points where patients can be involved in patient safety. At each workshop, the discussions were facilitated by researchers with experience of patient safety in primary care and involving people in research along with public contributors. At these events, notes were taken during discussions, memos written afterwards along with post‐it notes, diagrams and online brief survey for feedback on prototypes (eg written comments, emails and discussion notes).^54^, ^63^

### 2.5 Data analysis

The co‐design process was cyclical involving several rounds of participant engagement with patients, carers, GPs and pharmacists. As the co‐design approach is participatory in nature, notes were made during the co‐design sessions and fed back to the groups to gain real‐time feedback and check interpretation. After the workshops participants were sent a written copy of notes to reflect on and add to. The research team worked with the public contributor members to identify key touch points by analysing the discussion notes and feedback using a thematic approach.^62^, ^66^ The initial themes were discussed, amended and/or merged until final themes and sub‐themes were developed. This analysis and interpretation were then presented to participants at subsequent co‐design events to get further feedback to support the credibility of the findings. The other stakeholders commented on the prototype to reflect wider stakeholder views and enhance the final prototype.

## 3 RESULTS

The three workshops were attended by a total of 35 people (22 patients and/or carers, six GPs and seven community pharmacists [see Table 1]). All three workshops involved discussions about what patient safety in primary care means to them and the way in which patient and carer involvement in safety may be enhanced by identifying key touch points. The following themes represent a synthesis of findings from the patient, carer and health‐care professionals.

**TABLE 1:** Number and type of participants per co‐design workshop

Workshop	Patients and/or carers	GPs	Pharmacists
1	16	4	4
2	15	6	5
3	8	3	2

### 3.1 The role of communication in safety

The importance of clear communication (both written and verbal) about expectations and mechanisms to raise safety issues was described by patient and/or carer participants. Patient and/or carer participants raised that they could be confused about what was happening, when and what they should do if they were not sure what to do. This was particularly exemplified when they had to go between care settings or had conflicting information from different health‐care professionals. Similarly, health‐care professional participants discussed the need to support patients and make them aware of the types of information they might be best to prepare and that patients and/or carers would correct inaccurate or erroneous information in their records. This was identified as a key role for patients to be involved in their own patient safety. Furthermore, the timeliness and clarity of information were considered an important element of communication that could create an environment which could foster patient involvement in patient safety from participants in the workshops.

For many of the participants, patient transfers across care settings were considered a time when safety issues were more likely to occur and to be more acute. For example, several patient and/or carer participants raised their concern about going into hospital and being unsure about what information to bring and what to do when discharged from hospital with existing medications and that missing information raised technical safety issues.

### 3.2 The roles of responsibilities of the patient and carer for safety: key touch points

Across all the discussions about involving patients and carers in patient safety in primary care, there was an identified need to support patients to learn about the appropriate use of services and ways patients and carers could take responsibility for their safety by adopting a number of small steps (both technical and relational key touch points [see Table 2]). For example, carers discussed how they needed to keep track of information from different health‐care providers. One participant described how when attending an appointment with the person they cared for, getting them ready, organizing transport, arriving at the appointment in advance, getting the person into the surgery and then into the consultation was exhausting and often forgot something they wanted to discuss which could affect the patient's care.

**TABLE 2:** The relational and technical key touch points of involving patients and carers in patient safety

Relational key touch points of involving patients and carers in safety	Technical key touch points of involving patients and carers in safety
Communication	Medication issues (eg incorrect medicine, incorrect dose, medication given to the wrong person, drug interactions)
Trust	What constitutes safety in different settings (GP, pharmacy, in and out of hospital)
Relationship between patient, carers and health‐care professionals	Where is the most appropriate place to seek care (eg GP, pharmacy, 111, 999)
Uncertainty of disclosure of private information to receptionists	Incorrect or delay in receiving diagnosis
Timely and clear information	Patients keeping and checking correspondence
Support and empowerment for patients and carers to be involved in their safety	Patients having access to their electronic medical records and checking it for accuracy
PSG‐PC should be complimentary to work in primary care	

Carer and patient participants identified that key questions or prompts might be useful to have to discuss with the person they look after to discuss in advance of a consultation so that they can discuss relevant issues to raise during the consultation which the person might not be able to raise themselves or they may forget. An aide memoire was seen as a keyway of supporting carers before a consultation as well as remembering what happened afterwards, particularly if they were the main co‐ordinator of care for someone with complex conditions and multiple appointments with different care providers.

Workshop participants identified that it was not enough to feel empowered to speak up but that they need to know what processes were available to support them in a manner that would not threaten the patient‐health‐care professional relationship. It was agreed by all attending the workshops that education around which services were available and what was considered an appropriate use of them should be included in the PSG‐PC.

### 3.3 The importance of partnerships and relationships to foster patient and carer involvement in patient safety

The participants agreed the need for patients and carers to take some responsibility for patient safety but also acknowledged that this would be difficult to do in isolation without the partnership from the health‐care professionals (eg between patient and pharmacist or patient and GP). Similarly, the health‐care professionals described this as being part of their role to support patients to be involved in being active in their care and decision making to the extent that the patient or carer wanted. For example, health‐care professional participants described the role of differential diagnoses and the evolving nature of diagnosis was discussed as a potential safety issue.

It was also identified that health‐care professional participants often assumed that if patients do not improve or get worse that they will seek a further consultation but did not necessarily explicitly say this. Conversely, some patient participants identified that they would not want to ‘bother the GP’ if they had not improved. For the PSG‐PC, this related to patients or carers knowing explicitly what to do if they were concerned or if their symptoms did not improve, within what timeframe or if the symptoms worsened.

## 4 DISCUSSION

A co‐design approach was used to develop the PSG‐PC for patients and carers to support their partnership in primary care patient safety. Our findings suggest that the EBCD approach was helpful in engagement participants’ perspectives and experiences about patient safety in primary care. The main touch points identified to support patients and carers involvement in patient safety were either relational (eg communication) or technical (eg medication safety issues). These key touch points could be used to serve as structure to develop a range of initiatives for supporting patients to be involved in patient safety with a focus on the wider population who utilize primary care which adds to the wider patient safety literature which often focuses on individual elements.^67^ The findings of this project suggest that there is a need to support people more broadly in primary care and the PSG‐PC was designed with GPs, pharmacists, patients and carers to enable this. The discussion emphasized the importance of using appropriate services for consultations which has the potential to reduce inappropriate demand on general practice, although may increase appropriate demand on other services. Despite a growing evidence about the difference in patients and carers perspectives of their role in patient safety,^2^, ^6^, ^17^, ^68^ there remains a lack of tools and support that patients and carers to be involved in primary care and this study has developed the PSG‐PC to address this gap.

A core premise to incorporating patients and carers within the primary care patient safety agenda was supporting effective communication between patients and clinicians. By incorporating appropriate models of communication within the PSG‐PC (including learning from shared decision making,^69^ values clarification exercises,^70^ health literacy,^71^ priority setting^72^ and behaviour change^73^, ^74^) as well as contextual issues that influence patient‐clinician communication,^28^, ^69^ providing positive feedback^75^ and having challenging conversations (eg nonviolent communication strategies), the PSG‐PC aims to support the inclusion of patients and carers in addressing key areas patient safety.^76^ Participants identified that in order for patients and family members to be meaningfully involved in their safety in primary care as part of their wider of care, they must have information, tools and support to participate that is appropriate and understandable.^77^, ^78^ Similarly, this draws on a patient‐centred approach to patient safety to develop support for patients involvement in their own patient safety in primary care.^79^ This perspective may consider patient safety as a component of self‐care with self‐care being defined by the World Health Organization as ‘the ability of individuals, families and communities to promote health, prevent disease, maintain health can to top with illness and disability with or without the support of a health‐care provider’.^80^ By adopting a patient safety lense in which to consider, this should be embedded within a whole systems approach to patient safety that incorporates the role of policy, culture and leadership^78^ to understand the context in which implementation of the PSG‐PC would occur in line with the national patient safety strategy.^14^, ^15^ Furthermore, in the development of guides and toolkits to improve patient safety there is a need to understand their suitability and acceptability to patients, carers and clinicians as well as potential changes to everyday practise with an understanding of intended and unintended consequences that may occur.^78^ This approach may paradoxically disenfranchise health professionals whilst overburdening patients and thus undermine the initiative.^39^ Creating a common goal to enhance patient safety may be one way of moving away from an approach which may ascribe blame.^68^

Co‐design approaches involve working together from the beginning with the intended end users of the intervention.^50^, ^81^ In this study, the partnership between patients, carers, GPs, community pharmacists and researchers generated ideas and contributed to the development and refinement of the PSG‐PC prototypes. In order to do this, the co‐design approach enabled all stakeholders to share their experiences so that the intervention is based on their needs.^50^ This approach builds on previous studies which have adopted a co‐design approach and the literature of patient involvement in patient safety to move beyond identifying the problem to developing a prototype to address it.^28^, ^29^, ^39^, ^54^, ^67^ The participants of the co‐design events identified key questions that patients and carers may want to ask, information that needed to be signposted to, and the need to involve health‐care professionals. Furthermore, the new national patient safety syllabus focuses on providing a multi‐disciplinary approach to patient safety which focuses on training staff within the NHS band yet whilst underpinned by involving patients within the wider strategy it does not yet provide an explicit component for patients or carers.^14^ Involving health‐care professionals with tools aimed to support patients similarly reflects the wider research on self‐care and self‐management support. Systematic reviews of self‐management support of any condition in primary care indicate that a structured patient‐provider exchange is necessary in primary care.^82^ This structured exchange should include the one‐to‐one patient‐health‐care provider consultation, on‐going follow‐up and provision of self‐help materials.^82^

### 4.1 Strengths and limitations of the study

Adopting a co‐design approach was a key strength of this study as it moved the discussion from focus only patient experiences of safety to focus on using those experiences to identify actions that could be used to enhance patient involvement in patient safety. A major strength of the co‐design approach supports drawing out both participants’ explicit knowledge and tacit knowledge of their experiences of using or delivering services.^83^ However, one limitation maybe that design being led by patients and clinicians may miss serious harms or risks that are not known to them. To overcome this limitation, the research team worked with experts in patient safety and national patient groups as well as a scoping exercise of the current literature. To overcome this, the research team comprised of experts in primary care patient safety research and worked with national patient groups to ensure the study was empirically informed whilst supporting a normative, or emancipatory, approach to research.^84^, ^85^, ^86^ Furthermore, the development of the PSG‐PC was informed by the national James Lind Alliance Priority Setting Partnership that identified the need to support safe communication and a current paucity in support.^18^ This approach goes further than previous studies which have focused on patient experience data by focusing on developing actions from experience.^87^ In order to address potential power differences between participants, [RM] began each session discussing the importance of ‘different hats’ that people wear and that there was not a single ‘correct’ view as well as using different tools and techniques to ensure participants felt able to voice their opinion if they wanted to (eg using post‐it notes, feedback, and group discussions).

Primary care incorporates a range of health‐care professionals who do not always work directly together. To address differences in practice GPs, community pharmacists, patients and carers were recruited broadly so that they were from a range of practices. We identified common themes emerging from discussions sought wider stakeholder involvement as well identifying existing literature on patient safety in primary care and consultation models to make sure that the PSG‐PC was complimentary to practice.^88^, ^89^ One strength of the study is that we asked participants to consider in their discussions what would work in their practice to make sure that from the outset and throughout discussions that the PSG‐PC would be realistic and useful in practices alongside existing tools and interventions and to ensure that it did not duplicate anything that was currently used. One limitation maybe the additional working task of using the PSG‐PC for patients or carers and the approach adopted attempted to identify realistic support and flexible support that allows the PSG‐PC to be used in a responsive rather than prescriptive approach. Future work should include examining if the PSG‐PC is acceptable and feasible to use in routine practice and whether it places an unacceptable workload on to patients, carers or health‐care professionals. This process of stakeholder engagement and refinement meant that it gave participants time to reflect on discussions and sense checking with different stakeholders to make the PSG‐PC as acceptable as possible. However, this process took investment and time to arrange. One limitation of the study is that it only involved GPs and community pharmacists and did not involve other professionals (eg practice nurses, district nurses and physiotherapists) as it was beyond the capacity of this project. Also, this work focused on developing a PSG‐PC in English and in paper but future work is needed to identify how people from different communities may want to be involved in patient safety and to culturally adapt the PSG‐PC and engage with different digital formats (eg mobile phone applications).

### 4.2 Conclusion

The PSG‐PC has the ability to address national and international patient safety priorities by providing a tool to involve patients in their patient safety by providing an approach to collaboratively support patients to take responsibility of their care in partnership with health‐care professionals. Participants identified the technical and relational key touch points of involving patients and carers in patient safety in primary care and mechanisms through which patients and carers can be involved. Co‐designing the PSG‐PC to support patients and carers needs to be both informative as well as individually customizable.

## CONFLICT OF INTEREST

None to declare.

## AUTHOR CONTRIBUTIONS

RM led the design of project, data collection, analysis, interpretation, prototype development and refinement and drafted the manuscript. SC conceived the project. SC, AR and KG helped design the project. SC, AR, KG and SG collected data. All authors developed the analysis and interpretation and critically contributed to the prototype development and manuscript.

## Data Availability

Research data are not shared.

## References

[1] Allen D , Braithwaite J , Sandall J , Waring J . Towards a sociology of healthcare safety and quality. Sociol Health Illn. 2015;38(2):181‐197. 26679563 10.1111/1467-9566.12390

[2] Panesar SS , de Siva D , Carson‐Stevens A , et al. How safe is primary care? A systematic review. BMJ Qual Saf. 2015;25:1‐10. 10.1136/bmjqs-2015-004178 26715764

[3] Sheikh A , Panesar SS , Larizgoitia I , et al. Safer primary care for all – A global imperative. Lancet Glob Health. 2013;1(4):e182–e183. 10.1016/S2214-109X(13)70030-5 25104342

[4] Panagioti M , Khan K , Keers RN , et al. Prevalence, severity, and nature of preventable patient harm across medical care settings: systematic review and meta‐analysis. BMJ. 2019;366:l418. 10.1136/bmj.l4185 PMC6939648 31315828

[5] NHS England .Primary care services. https://www.england.nhs.uk/participation/get‐involved/how/primarycare/. Accessed August 01, 2020.

[6] Daker‐White G , Hays R , McSharry J , et al. Blame the patient, blame the doctor or blame the system? A meta‐synthesis of qualitative studies of patient safety in primary care. PLoS One. 2016;10:8. 10.1371/journal.pone.0128329 PMC4526558 26244494

[7] Vincent C . Patient safety. West Sussex: Wiley‐Blackwell 2010.

[8] Ricci‐Cabello I , Marsden KS , Avery AJ , et al. Patients evaluations of patient safety in English general practices: a cross‐sectional study. BJGP. 2017;67(660):e474‐e482. 10.3399/bjgp17X691085 PMC5565856 28583945

[9] Vincent C , Amalberti R . Safer Healthcare: strategies for the real world. Switzerland: Springer; 2016. 29465922

[10] Vosper H , Hignett S , Bowie P . Twelve tips for embedding human factors and ergonomics principles in healthcare. Med Teach. 2018;40(4):357‐363. 29126356 10.1080/0142159X.2017.1387240

[11] Vincent, Burnett, Carthey . The measurement and monitoring of safety. Health Foundation. 2013 http://www.health.org.uk/publications/the‐measurement‐and‐monitoring‐of‐safety/. Accessed December 12, 2019.

[12] Berwick D .Improving the safety of patients in England. 2013 https://www.gov.uk/government/uploads/system/uploads/attachment_data/file/226703/Berwick_Report.pdf. Accessed December 10, 2019.

[13] Francis R .Independent Inquiry into care provided by Mid Staffordshire NHS Foundation Trust January 2005 – March 2009. Vol I. 2010 https://assets.publishing.service.gov.uk/government/uploads/system/uploads/attachment_data/file/279109/0375_i.pdf . Accessed December 10, 2019.

[14] NHS England and NHS Improvement .The NHS Patient Safety Strategy: safer culture, safer systems, safer patients. 2019 https://improvement.nhs.uk/documents/5472/190708_Patient_Safety_Strategy_for_website_v4.pdf. Accessed December 10, 2019.

[15] NHS Improvement .Framework for involving patients in patient safety. 2020 https://engage.improvement.nhs.uk/policy‐strategy‐and‐delivery‐management/framework‐for‐involving‐patients‐in‐patient‐safety/user_uploads/200310‐draft‐framework‐for‐involving‐patients‐in‐patient‐safety.pdf. Accessed April 01, 2020.

[16] Idema RA , Allen S , Britton K , Gallagher TH . What do patients and relatives know about problems and failures in care? BMJ Qual Saf. 2012;21:198‐205. 10.1136/bmjqs-2011-000100 22178930

[17] Scott D , Jones D . Do older patients perception of safety highlight barriers that could make their care safer during organizational transfer. BMJ Qual Saf. 2012;21:112‐117. 10.1136/bmjqs-2011-000300 22069114

[18] Morris RL , Stocks SJ , Alam R , et al. Identifying primary care patient safety research priorities in the UK: a James Lind Alliance Priority Setting Partnership. BMJ Open. 2018;8:e020870. 10.1136/bmjopen-2017-020870 PMC5855454 29490970

[19] QC . High level guidance to support a shared view of quality in general practice. 2018 https://www.cqc.org.uk/sites/default/files/20180322_high‐level‐guidance‐to‐support‐shared‐view‐of‐quality‐in‐general‐practice.pdf. Accessed December 10, 2019.

[20] The Health Foundation . Evidence scan: levels of harm. 2014 http://www.health.org.uk/publications/levels‐of‐harm/. Accessed December 12, 2019.

[21] Aase L , Waring J , Schibevaag L . Researching quality in care transitions. Switzerland: Palgrave Macmillan; 2017.

[22] Wachter RM . Patient safety at ten: unmistakable progress, troubling gaps. Health Aff. 2010;29(1):165‐173. 10.1377/hlthaff.2009.0785 19952010

[23] Stavropoulou C , Doherty C , Tosey P . How effective are incident‐reporting systems for improving patient safety? A systematic literature review. Millbank Q. 2015;93(4):826‐866. 10.1111/1468-0009.12166 PMC4678941 26626987

[24] Campbell SM , Bell BG , Marsden K , et al. A patient safety toolkit for family practices. J Patient Saf. 2020;16(3):e182‐e186. 10.1097/PTS.0000000000000471 PMC7447126 29461334

[25] Parent R , Roy M , St‐Jeacques D . A systems‐based dynamic knowledge transfer capacity model. J Knowledge Manag. 2007;11:81‐93.

[26] Jensen CB . Sociology, systems and (patient) safety: knowledge translations in healthcare policy. Sociol Health Ill. 2008;30:309‐324. 10.1111/j.1467-9566.2007.01035.x 18290938

[27] Ward V , Smith S , House A , et al. Exploring knowledge exchange: a useful framework for practice and policy. Soc Sci Med. 2012;74:297‐304. 22014420 10.1016/j.socscimed.2011.09.021

[28] Rhodes P , Sanders C , Campbell S . Relationship continuity: when and why do primary care patients think it is safer? BJGP. 2016;64(629):e758‐e764. 10.3399/bjgp14X682825 PMC4240148 25452540

[29] Stocks SJ , Donnelly A , Esmail A , et al. Frequency and nature of potentially harmful preventable problems in primary care form the patient’s perspective with clinician review: a population‐level survey in Great Britain. BMJ Open. 2018;8:1–14. 10.1136/bmjopen-2017-020952 PMC6009615 29899057

[30] The Health Foundation . Person‐centred care made simple. 2014. http://www.health.org.uk/sites/health/files/PersonCentredCareMadeSimple.pdf. Accessed December 10, 2019.

[31] Rowley E , Wright N , Waring J , et al. Protocol for an exploration of knowledge sharing for improved discharge from a mental health ward. BMJ Open. 2014;4:e005176. 10.1136/bmjopen-2014-005176 PMC4185338 25273812

[32] De Wet C .An Overview of patient safety in primary care. 2012 https://www.nes.scot.nhs.uk/media/2075343/an‐overview‐of‐patient‐safety‐in‐primary‐care‐nov‐12.pdf. Accessed December 10, 2019.

[33] Born KB , Levinson W . Choosing wisely campaigns globally: a shared approach to taking the problem of overuse in healthcare. J Gen Fam Med. 2019;20(1):9‐12. 30631653 10.1002/jgf2.225 PMC6321837

[34] Alam R , Cheraghi‐Sohi S , Panagioti M . Managing diagnostic uncertainty in primary care: a systematic critical review. BMC Fam Prac. 2017;18:79. 10.1186/s12875-017-0650-0 PMC5545872 28784088

[35] Daker‐White G , Hays R , Esmail A , et al. MAXimising Involvement in MUltiMorbidity (MAXIMUM) in primary care: protocol for an observation and interview study of patients, GPs and other care providers to identify ways of reducing patient safety failures. BMJ Open. 2014;4:8. 10.1136/bmjopen-2014-005493 PMC4139641 25138807

[36] Jerak‐Zuiderant S . Certain uncertainties: modes of patient safety in healthcare. Soc Stud Sci. 2012;42:732‐752. 23189612 10.1177/0306312712448122

[37] Balogh E , Miller BT , Ball JR .Improving diagnosis in health care. Institute of Medicine. 2015 http://www.nationalacademies.org/hmd/Reports/2015/Improving‐Diagnosis‐in‐Healthcare.aspx. Accessed December 10, 2019. 26803862

[38] Kale MS , Korenstein D . Overdiagnosis in primary care: framing the problem and finding solutions. BMJ. 2018;362:k2820. 30108054 10.1136/bmj.k2820 PMC6889862

[39] Sutton E , Eborall H , Martin G . Patient Involvement in patient safety: current experiences, insights from the wider literature, promising opportunities? PMR. 2015;17(1):72‐89.

[40] Heavey E , Waring J , Brun AD , et al. Patients’ concepualizations of responsibility for understanding differing attributions in the context of patient safety. J Health Soc Behav. 2019;60:188‐203. 31113253 10.1177/0022146519849027

[41] Davis RE , Jacklin R , Sevdalis N , Vincent C . Patient involvement in patient safety: what factors influence patient participation and engagement? Health Expect. 2007;10:259‐267. 17678514 10.1111/j.1369-7625.2007.00450.x PMC5060404

[42] Davis RE , Sevdalis N , Vincent CA . Patient involvement in patient safety: how willing are patients to participate? BMJ Qual Saf. 2011;20:108‐114. 10.1136/bmjqs.2010.041871 21228083

[43] Dixon‐Woods M . Why is patient safety so hard? A selective review of ethnographic studies. J Health Serv Res Policy. 2010;2010:15. 10.1258/jhsrp.2009.009041 20075122

[44] Sheard L , Marsh C , Mills T , et al. . Using patient experience data to develop a patient experience toolkit to improve hospital care: a mixed‐methods study. Southampton: NIHR Journals Library; 2019. 31682393

[45] John Hopkins Medicine . The John Hopkins Hospital patient and family handbook. 2019 https://www.hopkinsmedicine.org/the_johns_hopkins_hospital/_docs/the‐johns‐hopkins‐hospital‐patient‐handbook.pdf. Accessed December 10, 2019.

[46] Danish Society for patient safety . Patient Handbook: a patient’s guide to safer hospital stay. 2016 http://arkiv.patientsikkerhed.dk/media/655535/patient_handbook2_.pdf. Accessed December 10, 2019.

[47] Wright J , Lawton R , O’Hara J , et al. . Improving patient safety through the involvement of patients: development and evaluation of novel interventions to engage patients in preventing patient safety incidents and protecting them against unintended harm. Southampton: NIHR Journals Library; 2016. 27763744

[48] Craig P , Dieppe P , Macintyre S , et al. Developing and evaluating complex interventions: the new Medical Research Council guidance. BMJ. 2008;337(sep29_1):a1655. 18824488 10.1136/bmj.a1655 PMC2769032

[49] Locock L , Robert G , Boaz A , et al. . Testing accelerated experience‐based co‐design: a qualitative study of using a national archive of patient experience narrative interviews to promote rapid patient‐centred service improvement. Southampton: NIHR Journals Library; 2014. 25642558

[50] Hjelmfors L , Stromberg A , Friedrichsen M , et al. Using co‐design to develop an intervention to improve communication about the heart failure trajectory and end‐of‐life care. BMC Palliat Care. 2018;17(85):1–10. 10.1186/s12904-018-0340-2 PMC5996457 29890974

[51] Bate P , Robert G . Experience‐based design: from redesigning the system around the patient to co‐designing services with the patient. Qual Saf Health Care. 2006;15(5):307–310. 17074863 10.1136/qshc.2005.016527 PMC2565809

[52] Wrede J , Voigt I , Bleidorn J , et al. Complex health care decisions with older patients in general practice: Patient‐centeredness and prioritization in consultations following a geriatric assessment. Patient Educ Couns. 2013;90(1):54‐60. 22884411 10.1016/j.pec.2012.07.015

[53] Bate P , Robert G . Bringing user experience to healthcare improvement. Oxford: Routledge; 2007.

[54] Hackett CL , Mulvale G , Miatello A . Co‐designing for quality: creating a user‐driven tool to improve quality in youth mental health services. Health Expect. 2018;21(6):1013‐1023. 29707865 10.1111/hex.12694 PMC6250867

[55] Miatell A , Mulvale G , Roussakis C . Using experiences to improve transitions from youth to adult mental health services: understanding perspectives and values of youth, caregivers and service providers for improving care process. J Mental Health Policy Econ. 2017;20:S25‐S26.

[56] Cranwell K , Polacsek M , McCann TV . Improving mental health service users with medical co‐morbidity transition between tertiary medical hospital and primary care services: a qualitative study. BMC Health Serv Res. 2016;16:302. 27456864 10.1186/s12913-016-1567-3 PMC4960840

[57] Bagley HJ , Short H , Harman NL , et al. A patient and public involvement (PPI) toolkit for meaningful and flexible involvement in clinical trials‐a work in progress. Res Involv Engagem. 2016;2:15. 29062516 10.1186/s40900-016-0029-8 PMC5611579

[58] INVOLVE . What is public involvement. http://www.invo.org.uk/find‐out‐more/what‐is‐public‐involvement‐in‐research‐2/. Accessed July 10, 2020.

[59] NHS England . NHS Five Year Forward View: Primary Care. https://www.england.nhs.uk/five‐year‐forward‐view/next‐steps‐on‐the‐nhs‐five‐year‐forward‐view/primary‐care/. Accessed August 1, 2020.

[60] INVOVLE . Payment and recognition of public involvement. 2019 https://www.invo.org.uk/resource‐centre/payment‐and‐recognition‐for‐public‐involvement/. Accessed December 10, 2019.

[61] HRA . What is public involvement in research. 2019 https://www.hra.nhs.uk/planning‐and‐improving‐research/best‐practice/public‐involvement/. Accessed December 10, 2019.

[62] Ziebland S , McPherson A . Making sense of qualitative data analysis: an introduction with illustrations from DIPEx (personal experiences of health and illness). Med Edu. 2006;40(5):405‐414. 10.1111/j.1365-2929.2006.02467.x 16635119

[63] Kuluski K , Ho JW , Cadel L , et al. An alternate level of care plan: co‐designing components of an intervention with patients, caregivers and providers to address delayed hospital discharge challenges. Health Expect. 2020;00:1‐11. 10.1111/hex.13094 PMC7696114 32602628

[64] Plain English Campaign . Crystal mark. http://www.plainenglish.co.uk/services/crystal‐mark.html. Accessed August 01, 2020.

[65] Staniszewska S , Brett J , Simera I , et al. GRIPP2 reporting checklists: tools to improve reporting of patient and public involvement in research. BMJ. 2017;3:13. 10.1186/s40900-017-0062-2 PMC5611595 29062538

[66] Patton MQ . Qualitative research and evaluation methods. London: SAGE Publishing 2015.

[67] Yu A , Flott K , Chainani N , Fontana G , Darzi A .Patient Safety 2030. London, UK: NIHR Imperial Patient Safety Translational Research Centre; 2016.

[68] Hor S , Godbold N , Collier A , Iedema R . Finding the patient in patient safety. Health. 2013;1‐17. 10.1177/1363459312472082 23349385

[69] Elwyn G , Frosch D , Thomson R , et al. Shared decision making: a model for clinical practice. J Gen Int Med. 2012;27(10):1361‐1367. 10.1007/s11606-012-2077-6 PMC3445676 22618581

[70] Protheroe J , Blakeman T , Bower P , et al. An intervention to promote patient participation and self‐management in long term conditions: development and feasibility testing. BMC Health Serv Res. 2010;10(1):206. 20630053 10.1186/1472-6963-10-206 PMC2912900

[71] WHO . Draft WHO European roadmap for implementation of health literacy initiatives through the life course. 2019 http://www.euro.who.int/__data/assets/pdf_file/0003/409125/69wd14e_Rev1_RoadmapOnHealthLiteracy_190323.pdf?ua=1. Accessed January 4, 2020.

[72] Wrede J , Voigt I , Bleidorm J . Complex health care decisions with older patients in general practice: Patient‐centeredness and prioritization in consultations following a geriatric assessment. Patient Educ Couns. 2013;90(1):54‐60. 22884411 10.1016/j.pec.2012.07.015

[73] Foy R , Overtveit J , Shekelle PG , et al. The role of theory in research to develop and evaluate the implementation of patient safety practices. BMJ Qual Saf. 2011;20:453‐459. 10.1136/bmjqs.2010.047993 21317181

[74] Dixon‐Woods M , Tarrant C , Willars J , et al. How will it work? A qualitative study of strategic stakeholders’ accounts of a patient safety initiative. Qual Saf Health Care. 2010;19:74‐78. 20172888 10.1136/qshc.2008.029504

[75] Visser MR , Smets EM , Oort FJ , De Haes HC . Stress, satisfaction and burnout among Dutch medical specialists. CMAJ. 2003;168(3):271‐275. 12566331 PMC140468

[76] Rosenberg M . Nonviolent communication: a language of life: life‐changing tools for healthy relationships. California: Puddle Dancer Press 2003.

[77] Elwyn G , Edwards A , Kinnersley P . Shared decision‐making in primary care: the neglected second half of the consultation. BJGP. 1999;49(443):477‐482. 10562751 PMC1313449

[78] Walters J , Turnock AC , Walters EG , Wood‐Baker R . Action plans with limited patient education only for exacerbations of chronic obstructive pulmonary disease. Cochrane Database Syst Rev. 2010;5:CD005074. 10.1002/14651858.CD005074.pub3 20464737

[79] Institute of Medicine . Crossing the quality chasm: a new health system for the 21^st^ century. National Academies Press; 2001. 25057539

[80] WHO . Self‐care health interventions. https://www.who.int/news‐room/fact‐sheets/detail/self‐care‐health‐interventions. Accessed August 01, 2020.

[81] Blackwell RW , Lowton K , Robert G , et al. Using experience‐based co‐design with older patients, their families and staff to improve palliative care experiences in the emergency department: a reflective critique on the process and outcomes. Int J Nurs Stud. 2017;68:83‐94. 28095347 10.1016/j.ijnurstu.2017.01.002

[82] Dineen‐Griffin S , Garcia‐Cardenas V , Williams K , Benrimoj SI . Helping patients help themselves: a systematic review of self‐management support strategies in primary health care practice. Plos One. 2019;14(8):e0220116. 31369582 10.1371/journal.pone.0220116 PMC6675068

[83] Papoulias C . Showing the unsayable: participatory visual approaches and the consultation of ‘patient experience’ in healthcare quality improvement. Health Care Anal. 2018;26:171‐188. 29038985 10.1007/s10728-017-0349-3 PMC5899993

[84] Tsianakas V , Robert G , Maben J , et al. Implementing patient‐centered cancer care: using experience‐based co‐design to improve patient experience in breast and lung cancer services. Support Care Cancer. 2012;20(11):2639‐2647. 22544223 10.1007/s00520-012-1470-3 PMC3461206

[85] Greenhalgh T , Hinton L , Finlay T . Frameworks for supporting patient and public involvement in research: systematic review and co‐design pilot. Health Expect. 2019;22(4):785‐801. 31012259 10.1111/hex.12888 PMC6737756

[86] Oliver K , Kothair A , Mays N . The dark side of coproduction: do the costs outweigh the benefits for health research? Health Res Policy Syst. 2019;17:33. 30922339 10.1186/s12961-019-0432-3 PMC6437844

[87] Israilov S . Cho HJ (2017) How co‐creation helped address hierarchy, overwhelmed patients, and conflicts of interest in health care quality and safety. AMA J Ethics. 2017;19(11):1139‐1145. 29168686 10.1001/journalofethics.2017.19.11.mhst1-1711

[88] Royal College of General Practitioners . The GP consultation in practice. 2010 http://www.rcgp.org.uk/training‐exams/~/media/Files/GP‐training‐and‐exams/Curriculum‐2012/RCGP‐Curriculum‐2‐01‐GP‐Consultation‐In‐Practice.ashx. Accessed December 10, 2019.

[89] Silverman J , Kurt S , Draper J . Skills for communicating with patients. Oxford: Radcliffe Publishing; 2005.

